# Examining access to care in clinical genomic research and medicine: Experiences from the CSER Consortium

**DOI:** 10.1017/cts.2021.855

**Published:** 2021-09-14

**Authors:** Amanda M. Gutierrez, Jill O. Robinson, Simon M. Outram, Hadley S. Smith, Stephanie A. Kraft, Katherine E. Donohue, Barbara B. Biesecker, Kyle B. Brothers, Flavia Chen, Benyam Hailu, Lucia A. Hindorff, Hannah Hoban, Rebecca L. Hsu, Sara J. Knight, Barbara A. Koenig, Katie L. Lewis, Kristen Hassmiller Lich, Julianne M. O’Daniel, Sonia Okuyama, Gail E. Tomlinson, Margaret Waltz, Benjamin S. Wilfond, Sara L. Ackerman, Mary A. Majumder

**Affiliations:** 1 Center for Medical Ethics and Health Policy, Baylor College of Medicine, Houston, TX, USA; 2 Program in Bioethics, University of California, San Francisco, San Francisco, CA, USA; 3 Treuman Katz Center for Pediatric Bioethics, Seattle Children’s Research Institute, Seattle, WA, USA; 4 Department of Pediatrics, University of Washington School of Medicine, Seattle, WA, USA; 5 Institute for Genomic Health, Icahn School of Medicine at Mount Sinai, New York, NY, USA; 6 RTI International, GenOmics, BiOinformatics and Translational Science, Washington DC, USA; 7 Department of Pediatrics, University of Louisville, Louisville, KY, USA; 8 Institute for Human Genetics, University of California, San Francisco, San Francisco, CA, USA; 9 National Institute of Minority Health and Health Disparities, National Institutes of Health, Bethesda, MD, USA; 10 Division of Genomic Medicine, National Human Genome Research Institute, National Institutes of Health, Bethesda, MD, USA; 11 Division of Epidemiology, Department of Internal Medicine, University of Utah School of Medicine, Salt Lake City, UT, USA; 12 University of California, San Francisco, San Francisco, CA, USA; 13 National Human Genome Research Institute, National Institutes of Health, Bethesda, MD, USA; 14 Department of Health Policy and Management, University of North Carolina Chapel Hill, Chapel Hill, NC, USA; 15 Department of Genetics, University of North Carolina Chapel Hill, Chapel Hill, NC, USA; 16 Division of Hematology-Oncology, Denver Health and Hospital Authority, Denver, CO, USA; 17 Division of Hematology-Oncology, Department of Pediatrics, University of Texas Health Science Center San Antonio, San Antonio, TX, USA; 18 Greehey Children’s Cancer Research Institute, University of Texas Health Science Center San Antonio, San Antonio, TX, USA; 19 Department of Social Medicine, University of North Carolina Chapel Hill, Chapel Hill, NC, USA; 20 Department of Social and Behavioral Sciences, University of California, San Francisco, San Francisco, CA, USA

**Keywords:** Genomics, genetics, genome sequencing, genetic testing, personalized medicine, precision medicine, access to care, access to genomic medicine, health policy, health equity, health disparities

## Abstract

**Introduction:**

Ensuring equitable access to health care is a widely agreed-upon goal in medicine, yet access to care is a multidimensional concept that is difficult to measure. Although frameworks exist to evaluate access to care generally, the concept of “access to genomic medicine” is largely unexplored and a clear framework for studying and addressing major dimensions is lacking.

**Methods:**

Comprised of seven clinical genomic research projects, the Clinical Sequencing Evidence-Generating Research consortium (CSER) presented opportunities to examine access to genomic medicine across diverse contexts. CSER emphasized engaging historically underrepresented and/or underserved populations. We used descriptive analysis of CSER participant survey data and qualitative case studies to explore anticipated and encountered access barriers and interventions to address them.

**Results:**

CSER’s enrolled population was largely lower income and racially and ethnically diverse, with many Spanish-preferring individuals. In surveys, less than a fifth (18.7%) of participants reported experiencing barriers to care. However, CSER project case studies revealed a more nuanced picture that highlighted the blurred boundary between access to genomic research and clinical care. Drawing on insights from CSER, we build on an existing framework to characterize the concept and dimensions of access to genomic medicine along with associated measures and improvement strategies.

**Conclusions:**

Our findings support adopting a broad conceptualization of access to care encompassing multiple dimensions, using mixed methods to study access issues, and investing in innovative improvement strategies. This conceptualization may inform clinical translation of other cutting-edge technologies and contribute to the promotion of equitable, effective, and efficient access to genomic medicine.

## Introduction

Advancing equity in genomic medicine requires consideration of access to care [1]. Leaders in genomics have expressed clear concern about inequitable access to genomic sequencing (GS), particularly among medically underserved groups [2,3] and cautioned that, without special attention to access barriers, moving GS technologies into the clinic could exacerbate those inequities [1,4,5]. A 2018 National Academies workshop that focused on disparities in access to genomic medicine began to characterize barriers in clinical contexts, including high out-of-pocket costs, workforce limitations, and lack of appropriate referrals [2]. Experts further highlighted the negative impacts of understudied disparities related to lower literacy levels, structural and interpersonal racism, and language and cultural differences on experiences with genomic medicine [2,6].

Interwoven with these barriers is the history of racism and exclusion in genetics and genomics, which have contributed to existing structural injustice in the USA health care system and research enterprise generally [2,6–9]. One legacy of this history is that populations of European descent and higher socioeconomic status are now disproportionately overrepresented in GS research and genomic databases, which has resulted in GS yielding less informative results and thus having lower clinical utility for patients from other groups [1,4,10–13]. To address these inequities, the field must focus on improving access to GS across underserved and underrepresented populations [1,4,10–12]. Importantly, access to genomics does not necessarily equate to utility of results, especially for historically excluded groups. In addition to removing barriers to access, the field has an ethical obligation to evaluate whether GS technologies provide clinical and social benefit for all populations.

Special attention to these intersecting access determinants is imperative to ensuring equitable implementation of genomics both within and outside clinical genomic research contexts. Fortunately, attention to barriers in accessing genomic medicine is advancing [2,3]. Access to care is included in the Genomic Medicine Integrative Research Framework intended to guide genomic medicine research [14], and the American College of Medical Genetics and Genomics (ACMG) has underlined that improved outcomes from genomic medicine depend on access to both testing and appropriate follow-up care [15,16]. However, the concept of “access to genomic medicine” lacks a clear, multidimensional framework, creating challenges to studying and addressing the full range of access barriers. Clinical genomic research can begin to provide insight into the concept of access to genomic medicine at the intersection of research and clinical care.

Here, we illustrate barriers to genomic medicine and improvement strategies using qualitative case studies and participant survey data from the Clinical Sequencing Evidence-Generating Research consortium (CSER). Drawing on the experience of CSER projects in different settings across the US, we delineate the important relationship between access to routine clinical care and access to clinical research, and the latter’s implications for access to genomic medicine. We build on an existing access to care framework [17] using insights from CSER to characterize dimensions of access to genomic medicine, along with associated measures and strategies for improving access, a conceptualization that can guide clinical research involving other cutting-edge technologies. Finally, we identify challenges and directions for future research using this framework, with a goal of promoting equitable, effective, and efficient access to genomic medicine.

## Materials and Methods

### The CSER Consortium

The CSER consortium included seven clinical research projects – six National Institutes of Health (NIH)-supported and one NIH-conducted – that aimed to broaden the evidence base for genomic medicine by studying the integration of GS into the care of diverse populations in various settings [18]. CSER projects began in May 2017 and were ongoing at the time of publication except for ClinSeq (2012–2017). Each project committed to a minimum of 60% of their study population meeting criteria for diversity [19], which focused on including racial and ethnic minority populations historically underrepresented in research and individuals residing in medically underserved areas [20].

### The Concept of “Access to Care”

Access to care is a broad, complex, and multidimensional concept encompassing many societal, contextual, and individual factors [17,21–24]. Contemporary frameworks for studying health care access include a range of financial and structural determinants, quality of care provided, and equity [17,25,26]. Here, we use the definition provided by Andersen and colleagues of access to care as “actual use of personal health services and everything that facilitates or impedes their use” [17]. This concept extends beyond health insurance coverage and proximity to providers [25] and aims at “getting to the right services at the right time to promote improved health outcomes” [17].

### Evaluating Access to Care in CSER

CSER collected participant data through surveys with measures harmonized across projects where possible [27–29]. Baseline surveys asked about access-related sociodemographic factors and about barriers to care directly through a two-item harmonized measure adapted from the Agency for Healthcare Research and Quality’s Medical Expenditure Panel Survey [30]. The CSER access to care measure asked whether respondents experienced barriers to accessing clinical care for themselves (adult patients) or for their children (pediatric patients) in the past year with multiple response options; respondents could select all that applied. We note that this measure was not specific to genomic medicine or designed to capture the complexity of health access experiences. Rather, it helped characterize one possible understanding of ‘medically underserved’ and suggested which general barriers were common in enrolled populations. Follow-up surveys administered five to seven months after return of GS results assessed whether clinical recommendations made based on results were followed.

Beyond surveys, CSER projects used additional methods to understand access issues among their specific populations. We provided structuring questions (Supplemental Material) to project investigators to develop their qualitative case studies [31]. We identified key themes in access barriers reported in case studies as well as calculated descriptive statistics of the harmonized access to care measure. Results are organized and grouped for analysis using Andersen et al.’s categories [17]. Barriers related to “beliefs (attitudes, values, and knowledge) and perceptions of need” included disliking doctors (to capture the residue of negative interactions with the health care system and mistrust at the individual level), not knowing where to get medical care, perceptions of not having time, waiting for a problem to resolve, and avoiding bad news. “Organizational” barriers included doctor availability, transport/travel limitations (related to distance to providers), and being refused services. “Financial” barriers included cost/affordability and insurance issues. “Social” barriers, an additional category which included language barriers, cultural discordance, literacy levels, and mistrust of the healthcare system at the community level, appeared in case studies but was not directly assessed by the harmonized measure. We report preliminary data collected through February 29, 2020.

## Results

### Contextual Factors and Participant Characteristics Related to Access to Care

While diverse geographically and clinically [2,6], all CSER projects targeted enrollment of racial and ethnic minority populations historically underrepresented in research and those considered medically underserved by either census tract or insurance status (Table 1). Relevant contextual factors [32–35] differing across the 13 states involved include Medicaid expansion status, uninsured rates, poverty levels, limited English proficiency (LEP), and population living in rural areas (Table 2). Notably, most projects were conducted in states with a higher proportion of their population being lower income relative to the national average. Demographically, those enrolled differed along individual factors related to access, including insurance and socioeconomic status, health literacy, preferred language, and self-reported race and ethnicity (Supplementary Material) [32–38]. Participants were largely lower income and racially and ethnically diverse, with many Spanish-preferring individuals, and health literacy levels were relatively high in most projects.

**Table 1.** CSER consortium projects – project background information

Study name^a^ (Lead institution)	Populations^b^	Sub-populations^b^	Care sites^b^
CHARM (Kaiser Permanente Northwest)	Adults (ages 18-49) at risk for hereditary cancer	Racial and ethnic minority, underserved by census tract, Medicaid/Medicare or uninsured, Spanish-preferring	Outpatient primary care clinics from two health systems: Kaiser Permanente Northwest (Portland, Oregon), Denver Health (a system of Federally Qualified Health Centers in Denver, Colorado)
ClinSeq (NIH/NHGRI)^c^	Adults, no specific phenotype	African American, Afro-Caribbean, African	National Institutes of Health Clinical Center (Bethesda, Maryland)
KidsCanSeq (Baylor College of Medicine)	Children with cancer and their parent(s)	Medically underserved, Hispanic/Latino, African American, Asian, Spanish-preferring	Academic and non-academic medical centers, outpatient clinics: Texas Children’s Hospital (Houston, Texas), MD Anderson Cancer Center (Houston, Texas), University of Texas Health Science Center at San Antonio (San Antonio, Texas), Children’s Hospital of San Antonio (San Antonio, Texas), Cook Children’s (Fort Worth, Texas), Vannie Cook Children’s Clinic (McAllen, Texas)
NCGENES 2 (University of North Carolina)	Children with suspected genetic conditions (developmental disabilities, dysmorphology, neuromuscular disorders)	African American, Hispanic/Latino, Medicaid or uninsured	Outpatient pediatric genetic and neurology clinics at academic medical centers; community hospital: University of North Carolina Chapel Hill (Chapel Hill, North Carolina), Mission Health (Asheville, North Carolina), East Carolina University (Greenville, North Carolina)
NYCKidSeq (Icahn School of Medicine at Mount Sinai & Montefiore Medical Center)	Children (ages 0-21) with suspected neurologic, immunologic, and cardiac genetic conditions	African American, Hispanic/Latino, Medicaid, Spanish-preferring	Academic medical centers, private practice: The Mount Sinai Hospital (Manhattan, New York), Mount Sinai Doctors Faculty Practice (Manhattan, New York), Mount Sinai Kravis Children’s Hospital (Manhattan, New York), Mount Sinai West Hospital (Manhattan, New York), Montefiore Medical Center (Bronx, New York), The Children’s Hospital at Montefiore (Bronx, New York)
P3EGS (University of California, San Francisco)	Infants and children with severe developmental disorders, with or without congenital anomalies (pediatric); women whose fetus has a structural anomaly (prenatal)	Underserved by census tract, Medicaid, Asian, Hispanic/Latino, African American	Academic medical center, outpatient clinics, neonatal intensive care unit; pediatric intensive care unit; community hospital:UCSF Benioff Children’s Hospital Mission Bay (San Francisco, California), UCSF Fetal Treatment Center (San Francisco, California), Zuckerberg San Francisco General Hospital (San Francisco, California), UCSF Benioff Children’s Hospital Oakland (Oakland, California), Fresno Community Health Center (Fresno, California)
SouthSeq (HudsonAlpha Institute for Biotechnology)	Newborns with suspected genetic conditions	African American, underserved rural	Academic and non-academic medical centers: Children’s Hospital of New Orleans (New Orleans, Louisiana), University of Alabama at Birmingham (Birmingham, Alabama), University of Louisville (Louisville, Kentucky), University of Mississippi Medical Center, Woman’s Hospital (Jackson, Mississippi)

CSER, Clinical Sequencing Evidence-Generating Research consortium; NIH, National Institutes of Health; NHGRI, National Human Genome Research Institute; CHARM, Cancer Health Assessments Reaching Many; NCGENES 2, North Carolina Clinical Genomic Evaluation by Next-generation Exome Sequencing 2; P3EGS, Program in Prenatal and Pediatric Genome Sequencing.

a All projects have study materials (including consent forms, education materials, and surveys) available in both English and Spanish, some of which are publicly available at https://cser-consortium.org/cser-research-materials.

b Adapted from Amendola et al. [18] and Goddard et al. [27].

c ClinSeq completed enrollment at the start of the extramural studies and thus did not assess the access to care or other non-access-related variables as in other CSER projects.

**Table 2.** CSER consortium projects – state background information

Study name (Lead institution)	State(s) involved	Medicaid expansion?^a^	Percent (%) of non-elderly adults without health insurance (2018 data)^b^	Percent (%) of population below 200% of the federal poverty line (2018 data)^c^	Percent (%) of population in rural areas (2018 data)^d^	Percent (%) of population with limited English proficiency^e^ (2015 data)^f^
United States average	–	–	9.2	30.4	14.0	8.4
CHARM (Kaiser Permanente Northwest)	Colorado	Yes	8.8	24.7	12.5	6.6
	Oregon	Yes	8.6	29.3	16.1	6.2
ClinSeq (NIH/NHGRI)	District of Columbia	Yes	3.5	27.4	0	4.8
	Maryland	Yes	6.9	20.9	2.5	6.4
	Virginia	Yes	10.1	24.5	12.2	5.6
KidsCanSeq (Baylor College of Medicine)	Texas	No	20.0	34.2	10.7	14.2
NCGENES 2 (University of North Carolina)	North Carolina	No	12.9	33.5	21.3	4.8
NYCKidSeq (Icahn School of Medicine at Mount Sinai & Montefiore Medical Center)	New York	Yes	6.2	29.7	7.0	13.4
P3EGS (University of California, San Francisco)	California	Yes	8.2	29.8	2.1	19.4
SouthSeq (HudsonAlpha Institute for Biotechnology)	Alabama	No	12.2	36.5	23.3	2.4
	Kentucky	Yes	6.6	36.1	41.0	2.1
	Louisiana	Yes	9.3	40.0	16.1	2.8
	Mississippi	No	14.5	41.3	53.4	1.6

CSER, Clinical Sequencing Evidence-Generating Research consortium; NIH, National Institutes of Health; NHGRI, National Human Genome Research Institute; CHARM, Cancer Health Assessments Reaching Many; NCGENES 2, North Carolina Clinical Genomic Evaluation by Next-generation Exome Sequencing 2; P3EGS, Program in Prenatal and Pediatric Genome Sequencing.

a Data from the Kaiser Family Foundation [39].

b Data from the Kaiser Family Foundation [40].

c Data from the Kaiser Family Foundation [41].

d Data from the USDA Economic Research Service [42].

e Data from LEP.gov [43].

f Limited English proficiency is defined as persons 5 years of age and older who speak English “less than very well” [44].

Descriptive analysis of responses to the harmonized access to care survey measure found that, of the five CSER projects that implemented the harmonized access to care measure, less than a fifth (18.7%) of participants reported experiencing any barrier to care within the past year (Table 3). About 85% of surveys sent to (or survey visits scheduled with) CSER participants across these five projects were completed. The most frequently reported type of barrier across all participants (n=374) was beliefs and perceptions of need (48.1%, *n* = 180), followed by organizational (27.0%, *n* = 101) and financial (24.9%, *n* = 93) barriers. The most frequently reported barriers and proportion of the enrolled population reporting any barriers differed by project.

**Table 3.** CSER consortium projects – preliminary data from the harmonized access to care survey measure (data through February 29, 2020)

Study name (Lead Institution)^a^	Surveys completed (%, *n*)	Respondents reporting care access issues in past 12 months^b^ (%, *n*)	Frequency of self-reported barriers to care^c,d^			
			Beliefs and perceptions of need (*n*, %)^e^	Organizational (n, %)^f^	Financial (*n*, %)^g^	Total barriers reported^b,c^ (*n*)
CHARM (Kaiser Permanente Northwest)	83.3% (763/916)	31.5% (240/763)	150 (63.3%)	39 (16.5%)	48 (20.3%)	237
KidsCanSeq (Baylor College of Medicine)	95.5% (296/310)	6.4% (19/296)	4 (22.2%)	1 (5.6%)	13 (72.2%)	18
NCGENES 2 (University of North Carolina)	95.0% (131/138)	13.0% (17/131)	6 (37.5%)	5 (31.3%)	5 (31.3%)	16
NYCKidSeq (Icahn School of Medicine at Mount Sinai & Montefiore Medical Center)	86.9% (446/513)^h^	14.1% (63/446)	8 (13.3%)	37 (61.7%)	15 (25.0%)	60
P3EGS (University of California, San Francisco)	75.3% (405/538)^h^ - Pediatric: 77.0% (281/365) - Prenatal: 71.7% (124/173)	10.4% (42/405) - Pediatric: 12.1% (34/281) - Prenatal: 6.5% (8/124)	12 (27.9%)	19 (44.2%)	12 (27.9%)	43
**Total across all CSER sites^b^**	**84.5% (2041/2415)**	**18.7% (381/2041)**	**180 (48.1%)**	**101 (27.0%)**	**93 (24.9%)**	**374**

CSER, Clinical Sequencing Evidence-Generating Research consortium; CHARM, Cancer Health Assessments Reaching Many; NCGENES 2, North Carolina Clinical Genomic Evaluation by Next-generation Exome Sequencing 2; P3EGS, Program in Prenatal and Pediatric Genome Sequencing.

a SouthSeq and ClinSeq did not assess the access to care variable.

b Does not include unavailable or missing data.

c Participants could select more than one barrier.

d Categories adapted from Andersen et al. [17].

e Beliefs and perceptions: disliking doctors, not knowing where to get medical care, perceptions of not having time, waiting for a problem to resolve, avoiding bad news.

f Organizational barriers: doctor availability, transport/travel limitations (related to distance to providers), being refused services.

g Financial barriers: cost/affordability, insurance issues.

h NYCKidSeq and P3EGS baseline surveys were administered only during study enrollment visits and not sent electronically as with other CSER projects, so this data reflects the number of completed survey study visits out of those scheduled.

### CSER Project Case Studies Related to Access to Care

The following case studies converged on two findings related to measuring access to genomics. First, despite only a minority of CSER participants reporting experiences of listed barriers via survey, projects reported a wide range of anticipated and encountered access barriers, suggesting the quantitative measures used did not comprehensively capture access issues. Second, general barriers to health care created obstacles to participants’ access to genomic services, and these barriers served as both an obstacle to and motivator for GS research enrollment, highlighting the complex relationship between access to research and clinical care. Table 4 provides an overview of barriers and strategies to evaluate and mitigate them drawn from case studies.

**Table 4.** Access barriers and barrier evaluation and mitigation strategies identified in CSER project case studies

Study name (Lead Institution)	Anticipated and encountered barriers to care^a^	Strategies to evaluate and mitigate barriers to care
CHARM (Kaiser Permanente Northwest)	Financial: - Cost/affordability issues - High uninsured rate - Limited referral options due to patient having public or no insurance - Disruptions in care (e.g., even with discount program, lapses in enrollment can create barriers to care) - Patient lack of primary care or incomplete family history impeding identification of indications for services Organizational: - Patient/provider time limitations - Transport/travel limitations - Referral-related challenges for genetic services (counseling, testing, etc.) - Lack of clinician knowledge about/ability to navigate referral options Social: - Lower literacy levels	- Manage informed consent, saliva sample collection by mail and results disclosure by phone. - Offer streamlined online process for cancer risk assessment and standardized collection of family history. - Evaluate the Accessible, Relational, Inclusive and Actionable (ARIA) model for genetic counseling. - Compile list of community resources for patients. - Conduct interviews to better understand referral barriers and inform planning (e.g., make the case for bringing more genetic services in-house at Federally Qualified Health Centers).
ClinSeq (NIH/NHGRI)	Beliefs and perceptions: - Low awareness of research/genetics - Mistrust of researchers and health care system Financial: - Cost/affordability issues Organizational: - Patient time limitations - Transport/travel limitations	- Use a recruiter from similar demographic background as underrepresented and/or underserved groups for in person recruitment. - Offer personalized/tailored recruitment. - Conduct focus groups to inform strategies to mitigate barriers to participation in clinical genomic research. - Develop targeted recruitment materials for underrepresented and/or underserved groups. - Return value to participants in the form of personal sequencing and other individual results.
KidsCanSeq (Baylor College of Medicine)	Financial: - Cost/affordability issues - High uninsured rate - Provider limitations due to patient having public insurance Social: - Language barriers/limited English proficiency - Cultural discordance between patients/parents/participants and clinicians/researchers - Lower literacy levels Organizational: - Access barriers to follow-up testing and care	- Pilot test Spanish surveys to improve wording for language accessibility. - Employ bilingual staff at each clinical site. - Use trained medical interpreters to help communicate genomic sequencing results to families. - Assess Spanish-preferring participants’ perceptions of and satisfaction with Spanish interpretation. - Assess barriers to follow-up testing and care via interviews and open-ended survey items.
NCGENES 2 (University of North Carolina)	Financial: - Cost/affordability issues - High uninsured rate Social: - Low levels of active caregiver engagement - Mistrust of health care system Organizational: - Parent/family time limitations - Transport/travel limitations	- Employ recruiters from similar demographics. - Recruit representative community stakeholder team to guide materials development, recruitment methods, and provide insight throughout. - Develop a plain-language pre-visit preparatory booklet with question prompt list through stakeholders’ input to support caregiver efficacy and engagement in child’s pediatric specialty appointments. - Use mixed methods (transcript analysis, surveys of caregivers and providers) to capture the effect of the preparatory materials on promoting active engagement of medically underserved patients. - Use surveys to assess caregiver experience, and understanding. - Use algorithms in clinical and claims data to identify patients who might benefit from but are not currently receiving genetic testing.
NYCKidSeq (Icahn School of Medicine at Mount Sinai & Montefiore Medical Center)	Financial: - Insurance denials of coverage for genetic panel testing Organizational: - Limited access to referred specialists and specialty care - Doctor unavailable Social: - Language barriers/limited English proficiency - Language discordance with clinicians	- Develop a novel communication tool, Genomic Understanding, Information and Awareness (GUÍA) that allows providers to create a visual and narrative guide for personalized return of results in English or Spanish via a web-based platform. - Test GUÍA to facilitate the return of genomic sequencing results in English and Spanish.
P3EGS (University of California, San Francisco)	Financial: - Transport/travel limitations - Cost/affordability issues Organizational: - Parent/family time limitations - Disparities in access to prenatal specialty care - Some patients unable to follow through with referrals	- Conduct interviews with families to explore full range of barriers to care (medical and non-medical support services). - Explore referral/enrollment barriers by conducting interviews with clinicians who refer patients for genetics consultation and work with medically underserved patients. - Offer virtual clinic appointments.
SouthSeq (HudsonAlpha Institute for Biotechnology)	Financial: - Cost/affordability issues Organizational: - Parent/family time limitations - Transport/travel limitations - Lack of social and clinical services in rural communities	- Conduct interviews with clinicians and families to understand barriers and facilitators to accessing comprehensive genomic health services for newborns, and gaps in support. - Use of engagement studios to tailor recruitment and counseling materials to the needs of families. - Literacy expert review of written study materials and revision of materials using plain language.

CSER, Clinical Sequencing Evidence-Generating Research consortium; NIH, National Institutes of Health; NHGRI, National Human Genome Research Institute; CHARM, Cancer Health Assessments Reaching Many; NCGENES 2, North Carolina Clinical Genomic Evaluation by Next-generation Exome Sequencing 2; P3EGS, Program in Prenatal and Pediatric Genome Sequencing; ARIA, Accessible, Relational, Inclusive and Actionable; GUÍA: Genomic Understanding, Information and Awareness.

a Categories adapted from Andersen et al. [17]: Beliefs and perceptions: attitudes, values, knowledge, perception of importance/magnitude of health issue. Social: education, occupation, race and ethnicity, social networks. Financial: insurance, cost, cost-sharing. Organizational: nature of sources of care, transportation, travel time, waiting time.

#### CHARM

The Cancer Health Assessments Reaching Many (CHARM) study aimed to increase access to genetic testing for hereditary cancer syndromes in low-income, low-education, and minority, adult populations, including LEP Spanish-preferring individuals. One CHARM site, Denver Health, is an integrated health system which includes nine Federally Qualified Health Centers (FQHCs) where 75% of patients are publicly insured or uninsured. Specialized care such as genomic medicine can be limited in safety net institutions like Denver Health [2]. Historically, Denver Health has referred patients who need genetic testing to outside institutions that provide limited access for Medicaid-covered or uninsured individuals. Denver Health offers uninsured patients an income-based sliding scale discount program that must be renewed annually; patients cannot complete a scheduled visit if their enrollment in the program has lapsed. In such instances, many participants selected “the doctor was not available to see me” on the CSER access to care measure, though this may not capture the experienced barrier.

To overcome barriers, CHARM offered a streamlined online process for risk assessment and informed consent, saliva sample collection by mail, and results disclosure by phone. Patient-facing materials were designed with an emphasis on literacy, and the study is evaluating a literacy-focused genetic counselling approach that uses evidence-based strategies for effective communication, such as avoiding jargon and using teach-back to assess comprehension through the Accessible, Relational, Inclusive and Actionable (ARIA) model of genetic counseling [45–47]. Through interviews, CHARM explored differences in who was referred for testing. Lack of referral can be due to patients not receiving regular prevention-focused primary care, incomplete family history precluding identification of testing need, or clinician lack of knowledge about (or inability to navigate) limited referral options. Preliminary data suggest that participants lacked access to genetic testing outside of CHARM, which was often cited as a motivation to enroll. Encouragingly, Denver Health’s participation in CHARM reduced organizational and structural barriers, including standardizing collection of family history in electronic health records, standardizing cancer risk assessment in the health record problem list, compiling community resources available for patients, and most importantly, pushing to offer GS inhouse.

#### CLINSEQ

An NIH-conducted clinical GS cohort study, the ClinSeq study recruited a primarily Black American cohort over five years (2012–2017), which consists of 467 largely healthy individuals who self-identify as African, African American, or Afro-Caribbean [48]. The lengthy recruitment time reflects multiple access issues, some of which were identified when the recruiter captured reasons for not enrolling in the study. Examples include logistics such as money, distance from the NIH, and work hours that overlapped with recruitment times, skepticism about the NIH, mistrust, and not knowing much about clinical research and/or genetics. These access issues remained throughout the enrollment period despite specific recruitment strategies designed to mitigate them. Strategies to facilitate recruitment of African-descended participants to genetics research included targeting recruitment materials [49], using a recruiter with similar demographic characteristics [50], offering interactive personalized/tailored recruitment [51], and returning personal sequencing results [52]. These strategies did promote community awareness of the study, which in turn increased enrollment, as participants reported being encouraged to enroll by someone they trusted or someone they knew who was enrolled.

The recruiter reported that participants “latched on” to the opportunity for a cardiac checkup offered at baseline. Those who enrolled were often motivated to learn information about their personal health (49%) or family members’ health (33%). Most participants had realistic expectations of sequencing and high levels of optimism, openness, and resilience. Our experience suggests that groups who have faced historical discrimination and exploitation in scientific research, and thus have reasons to mistrust researchers, may be more skeptical when weighing the risks and benefits of participating. Focus group findings among participants (unpublished results) revealed residual feelings of mistrust that were a barrier to participation earlier in recruitment.

#### KidsCanSeq

The Texas KidsCanSeq project studied the utility of GS in the case of pediatric cancer patients, enrolling patients and their parents, with six sites across the state. Texas is notably the largest state to decline participation in Medicaid expansion under the Affordable Care Act, and state requirements for Medicaid eligibility are restrictive [53,54]. Almost one-third of the 297 enrolled KidsCanSeq parents were uninsured, though preliminary data show few parents reported barriers to accessing care for their child in the past year, and most cited affordability issues and wanting to see if the problem would resolve on its own. KidsCanSeq parents and children differed in uninsured rates (35% vs 6%, respectively) and Medicaid/CHIP participation (20% vs 49%, respectively). KidsCanSeq included project-specific open-ended survey questions to assess barriers to follow-up care for participants and family members given that gaps in insurance coverage affect cascade testing [55] and accessing follow-up testing was an issue in the prior CSER study BASIC3 [56]. KidsCanSeq was also in the process of interviewing a subgroup of participants with significant findings about their experiences accessing recommended follow-up testing and care.

Nearly a third of Hispanic/Latino KidsCanSeq participants preferred to speak Spanish as their primary language, and more than half of those Spanish-preferring participants were LEP. To accommodate the large Spanish-preferring LEP population, KidsCanSeq had bilingual study staff at each clinical site and used trained medical interpreters to help communicate GS results to families. KidsCanSeq was also in the process of assessing Spanish-preferring LEP participants’ perceptions of the quality of and satisfaction with communication from interpreters. Additionally, at the clinical site on the US-Mexico border, the research team received feedback early into data collection that answering the CSER access to care measure was challenging. A pretest of the survey with Spanish-speakers outside of the study showed some understood this question as blaming them for not seeking care for their child. Ultimately, the substance of the question was not modified given the importance of harmonized measures and limited nature of the feedback, but a brief description was added to describe the purpose as understanding any issues experienced.

#### NCGENES 2

The North Carolina Clinical Genomic Evaluation by Next-generation Exome Sequencing 2 (NCGENES 2) study explored the use of genomic technologies in children with suspected genetic conditions recruited from three sites across North Carolina. Each site differs in population, ranging from more racially and ethnically diverse (50% other than White, non-Hispanic in University of North Carolina Pediatric Specialty clinics and 60% in East Carolina University), to a population that is predominately White (85%) and largely covered by Medicaid (70%; Mission, in Appalachia). Overall, 20% of North Carolina residents are enrolled in Medicaid, more than half (53%) of whom are children. NCGENES 2 explored socioeconomic factors, trust in providers, and distance from the clinic as participant enrollment and retention barriers. We recruited a caregiver stakeholder team, representative of study demographics, to guide study material development, recruitment and dissemination, and provide insight for data analysis. As participants often skipped income-related questions, NCGENES 2 added project-specific survey items about caregivers’ financial stress related to their child’s condition or treatments, including work missed and financial control.

To examine factors affecting the patient–provider relationship, with caregiver stakeholders’ input we developed a plain-language pre-visit preparatory booklet with question prompt lists to guide expectations and support caregiver self-efficacy for active engagement in pediatric specialty appointments [57–60]. Participants were randomized to receive the booklet or not and engagement was captured through a qualitative coding scheme applied to transcripts of recorded visits and survey items about questions asked and answered, understanding, and their trust in their physician and the medical field. The study also involved using machine learning and other analytical approaches to characterize the diagnostic odyssey among pediatric patients obtaining genetic testing and discriminating it from matched pediatric patients not appropriate for genetic testing based on their prior health services utilization and diagnostic codes in clinical and/or claims data. Such algorithms will be used in clinical and claims data to identify patients who might benefit from but are not currently receiving genetic testing.

#### NYCKidSeq

The NYCKidSeq study aimed to assess the clinical utility of genomic medicine in three broad areas of pediatric disorders. Recruitment primarily targeted underserved populations in Harlem and the Bronx. Of the 446 currently enrolled families, 49% identified as Hispanic/Latino and 22% as Black/African American. Over half of parents spoke a language other than English (58%) and 22% of study visits were conducted in Spanish. Despite the high number of Spanish-preferring participants, only 17% stated they preferred to speak a language other than English with their health provider. This was likely due to the limited number of providers who speak Spanish fluently, which itself can be an access barrier. Also, while study materials were available in Spanish, the team has noted that language barriers impacted access to support groups, which were typically conducted in English.

Most participants were insured through Medicaid (65%) and noted few insurance coverage difficulties. Nevertheless, 14% of families reported barriers via the access to care survey measure, with nearly a quarter citing affordability issues. The other barrier frequently cited was doctor unavailability, suggesting that even with Medicaid expansion, availability of pediatric specialists such as neurologists, cardiologists, and immunologists – specialties from which NYCKidSeq receives the most referrals – is limited. Anecdotally, the study team noted that many participants were referred to NYCKidSeq due to insurance denials of genetic panel testing ordered by specialists, indicating that genomics research is being used as a means of accessing needed clinical care. The study included testing a novel communication tool, Genomic Understanding, Information and Awareness (GUÍA), to facilitate the return of GS results [61,62]. GUÍA allows providers to create a visual and narrative guide for personalized return of results and relevant clinical information, in English or Spanish, via a web-based platform.

#### P3EGS

UCSF’s Program in Prenatal and Pediatric Genomic Sequencing (P3EGS) studied the utility of exome sequencing for children with suspected genetic conditions and pregnant women with fetal anomalies. Of the 538 participants (365 pediatric; 173 prenatal) enrolled, most were covered by Medi-Cal, California’s Medicaid program. Three access barriers were identified in survey responses and in-depth interviews with families. First, a high proportion of participants faced challenges related to the costs, travel time, and loss of work associated with clinic visits. Second, many families enrolled in P3EGS to obtain a test that would otherwise be unavailable to them without paying out of pocket. Third, families often enrolled with the hope that a genetic diagnosis would enhance their child’s eligibility for community-based supportive services. These findings indicate that access concerns are relevant both upstream and downstream of genetic testing. Additionally, contextual analysis suggested many eligible families did not enroll in the study because of access barriers.

To explore enrollment barriers in the pediatric arm, P3EGS conducted interviews with clinicians who referred patients for genetics consultation at UCSF and worked with a high proportion of medically underserved patients. They reported that many patients who were referred to genetics “fall through the cracks,” i.e., were not ultimately scheduled for or failed to attend an appointment. In the prenatal arm, demographic characteristics indicated enrollment barriers among low-income patients. Specifically, Medi-Cal pays for over 50% of births in California but only 20% of P3EGS prenatal participants were on Medi-Cal (compared with 86% in the pediatric arm). Prenatal participants also had higher self-reported income and were less racially and ethnically diverse than the overall population of women giving birth in California. Since prior genetic testing is an entry criterion for fetal exome sequencing, and depends on prior ultrasound and amniocentesis, disparities in access to fetal ultrasound and demographic differences in amniocentesis acceptance [63] may be influencing referral and enrollment.

#### SouthSeq

The SouthSeq project studied GS for diagnosis of genetic conditions in newborns in Neonatal Intensive Care Units (NICUs) across four Southern states. SouthSeq included a two-arm noninferiority trial that compared return of sequencing results by genetic counselors to return by non-genetics health professionals, such as neonatologists, who had been trained and supported by genetic counselors. Of the 224 newborns enrolled, most met CSER’s criteria for diversity (73%), including nearly half who were non-White (47%). Only two of four SouthSeq states have expanded Medicaid, and several were considering or have added Medicaid work requirements, an issue of particular concern for SouthSeq as parent work schedules may have impacted access to newborn care. Early in the project, SouthSeq used engagement studios to adapt materials for the needs of families, particularly those from groups underrepresented in biomedical research, and involved literacy experts to ensure study materials were written in plain language.

SouthSeq conducted qualitative interviews with clinicians and families to understand barriers and facilitators to accessing comprehensive genomic health services for newborns, and parental and family needs for and gaps in support that occurred during the NICU-to-home transition. SouthSeq found that, while GS may offer clarification about a genetic basis for their baby’s condition and provide critical guidance relevant to medical and non-medical care, one key challenge faced by families was the tremendous increase in parental responsibilities during this transition. For SouthSeq families residing in rural areas, the distance between home and the NICU created additional burdens including travel expenses and challenges supporting other children at home. The lack of social and clinical services in rural communities also reduced support for families after transition. Family support needs at home varied for parents, and possibly their other children, with some leaving the NICU with a baby who had many special needs and others without their baby who had passed away. At the time of return of results, approximately 66% of babies had been discharged or were deceased.

## Discussion

Survey data and case studies from CSER provide rich insight into how to conceptualize, evaluate, and improve access to genomic medicine. CSER experiences delineate an important relationship between access to clinical care and genomic research: participation in clinical GS research is a pathway to genomic medicine (as all enrolled patients, or at least half in the case of some clinical trials, may be sequenced). This pathway through genomic research participation facilitates access for some individuals who may not otherwise be able to access genomic medicine directly. However, though GS and other components of genomic medicine such as genetic counseling may be provided in a research study as in CSER, genetic results may also indicate a need for clinical (and non-clinical) follow-up, including enhanced surveillance and cascade testing. In such cases, research participants and their family members may have difficulty accessing these services [2,64], often for reasons similar to those preventing them from directly accessing genomic medicine in the first place. Further, CSER’s experiences have also uncovered how existing clinical GS research likely omits individuals who already experience significant barriers to care [65], exacerbating inequities at earlier stages of the research. Individuals with already limited access to basic health care may not prioritize research participation or find their way to sites that conduct clinical research [9], or they may not be referred [4]. Thus, general health care access is ordinarily necessary for access to genomic medicine, either directly as part of clinical care or via research participation.

### Access to Genomic Medicine Conceptualization and Dimensions

**Fig. 1.**
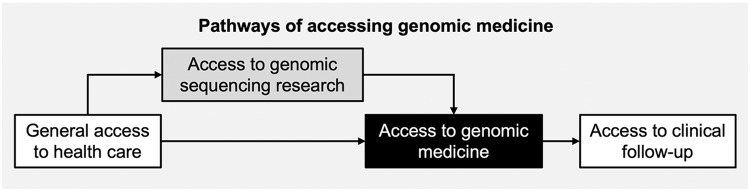
The relationship between access to health care, genomic research, and genomic medicine.

Fig. 1 illustrates the two main pathways of accessing genomic medicine identified in CSER. This entanglement of research with clinical genomics complicates our understanding of how to improve access to genomic medicine. However, using lessons learned from CSER along with Andersen and colleagues’ definition and dimensions of access to care [17], we can begin to characterize dimensions of access to genomic medicine. Table 5 outlines a conceptualization of access to genomic medicine and possible evaluation and improvement strategies along five dimensions: potential, realized, equitable, effective, and efficient access [17]. We recommend that any access to genomic medicine framework include the clinical GS research pathway and use mixed methods to comprehensively capture the access landscape. Though developed in a genomic context, this conceptualization can be applied to research involving other cutting-edge technologies. In the following section, we discuss lessons learned in CSER under each access dimension.

**Table 5.** Dimensions of access to genomic medicine and possible evaluation and improvement strategies (adapted from Andersen et al. [17])

Dimension	Definition	Possible evaluation and improvement strategies
Potential access	Captures conditions and factors that facilitate or impede receipt of genomic medicine	- Utilize measures that capture both contextual-level (e.g., health system organization, community characteristics) and individual-level factors (e.g., demographic, social, attitudinal). - Remove barriers to appropriate clinical genomic medicine and genomic research participation. - Limit inappropriate use of clinical services. - Advocate for providers and insurers to pay for enabling services that address clinical care barriers.
Realized access	Captures actual receipt of genomic medicine including genomic sequencing and recommended follow-up care	- Utilize both objective (evidence of receipt of care) and subjective measures (satisfaction with quality of care, including quality of interactions with providers and interpreters, where applicable). - Evaluate access to genomic medicine relative to patients’ perception of need (and/or need determined by external standard of appropriateness). - Make efforts to align access with perceived need.
Equitable access	Achieved when realized access to genomic medicine correlates with need and other characteristics unrelated to need (e.g., race and ethnicity, residence, insurance, income) are not determinants	- Advocate for changes to public and payer policies to minimize disparities. - Engage communities that face racism and other forms of social oppression and disadvantage. - Set targets for research enrollment and delivery of genomic medicine to underserved populations. - Develop and test interventions to address barriers known to disproportionately impact underserved populations. - Examine the relationship between direct or proxy measures of need for genomic medicine and measures of realized access to genomic medicine.
Effective access	Achieved when there is timely use of genomic medicine to achieve the best possible outcomes	- Examine the effect of potential and realized access on outcomes. - Support confidence in interpretation of genomic sequencing results across populations. - Utilize patient (or patient proxy)-reported outcomes. - Ensure outcomes are not compromised by language, cultural, and literacy barriers.
Efficient access	Achieved when genomic medicine yields the greatest level of health and well-being possible given resource constraints	- Examine the effect of potential and realized access on outcomes taking account of cost of producing outcomes. - Conduct cost-effectiveness analysis. - Situate cost-focused analyses in abroader context. - Elicit and integrate patients’ perspectives of utility in relation to burden and cost to patients of obtaining care. - Evaluate whether efforts to increase access to genomic medicine lead to elimination of disparities in the utility of genomic sequencing across ancestry groups.

### Lessons Learned and Recommendations

#### Potential access to genomic medicine

Through CSER’s efforts to evaluate the factors impacting potential access to genomic medicine, we demonstrated mixed methods are ideal for capturing individual and contextual access issues in both a statistically demonstrable and contextually rich way. We found that while preliminary data from CSER surveys suggested general care access barriers were uncommon among enrolled populations, the case studies contributed to a more nuanced picture and highlighted qualitative findings describing significant barriers and their impact. Thus, surveys systematically captured some relevant information, while qualitative methods such as interviews complementarily helped uncover complexity [66] and provided insight into the “why and how” of barriers, which “is necessary for developing and implementing interventions to reduce disparities” [67]. Additionally, more longitudinal mixed methods research is necessary to better understand access to recommended follow-up care such as cascade testing or enhanced surveillance [55,68–70], with attention to situations when use of clinical services may be inappropriate. In terms of addressing potential access barriers, CSER projects were unable to mitigate all the logistical barriers to general health care experienced by both participants and people who were altogether unable to access the clinical care sites conducting the research. Going forward, genomic research programs should build in measures to help lessen these barriers such as by utilizing telemedicine, providing transportation, compensating for travel costs and work missed, and integrating support services like offering childcare. While having genomic research programs fund these measures will likely increase access to genomic research, barriers to potential and realized access will remain once the research phase ends. Advocacy for more health care providers and insurers (especially Medicaid plans) to pay for enabling services that address these barriers in the clinical context is therefore also critical [2,71]. Future research should also aim to expand efforts to evaluate strategies for increasing access to GS and related services in rural community health centers, FQHCs, and other health care providers outside of higher-resourced academic medical centers.

#### Realized access to genomic medicine

Our work in CSER suggests that enrollment in GS research is a facilitator of realized access to genomic medicine. Our preliminary findings showed low-income patients may enroll in GS research to obtain a test that higher resourced patients can obtain clinically. Clinical context shapes research enrollment rates. Parents of pediatric cancer patients often enroll in GS research in hopes of finding a cause of or better treatment for their child’s cancer [72,73]. For many families of children with undiagnosed conditions, the possibility that a genetic diagnosis could help procure therapeutic and educational services provides a strong impetus for research participation, particularly for those dependent on publicly funded services. Unfortunately, GS information does not necessarily lead to access to these services, and for some, a genetic diagnosis could bring new barriers. For example, if a child is categorized as special needs by the education system, the family will need additional services to either support their child or contest the designation. Further, enrollment and continued follow-up in clinical GS research is itself a marker of access to, knowledge of, and success in navigating the US healthcare system. Even with intensive efforts to reach underserved populations, enrolling those facing significant access barriers to better understand and mitigate those very barriers remains challenging and issues of mistrust may persist. These experiences suggest the need to further assess the ethical implications of the blurred distinction between research and clinical care [74,75] with a focus on equity and earning trust. Strategies to improve realized access should aim to align access with perceived need, which should be evaluated using both objective measures (focused on utilization, e.g., a visit with a relevant care provider) and subjective measures (focused on satisfaction, e.g., convenience factors like travel and wait times; provider courtesy and concern; overall satisfaction with an episode of care) [23].

#### Equitable access to genomic medicine

Progress around equitable access to genomic medicine will depend on expanding the insured population, changing payer policies, and implementing state and federal legislation to broadly improve genetic testing and counseling [76–79]. Differences in Medicaid eligibility across states have been shown to affect health care disparities [80–82]. However, having health insurance does not necessarily enable use of cutting-edge technologies like clinical GS, as some public insurers do not cover GS in circumstances that private insurers do [83–85]. Also relevant are other insurance arrangements, such as cost sharing for needed GS and follow-up care or networks limiting access to specialists who offer genomic medicine. Our findings underscore how differences in payer policies and clinical contexts prevent a straightforward understanding of the impact of insurance on access to cutting-edge technologies, and that changes to these policies are necessary for minimizing disparities. Further, equitable access entails not only engaging underserved communities and setting diversity targets for research enrollment (in the case of CSER projects, at least 60% of the study population) and delivery of genomic medicine, but also developing and testing interventions like the ARIA Model [47] and GUÍA tool [61] to address literacy, language, and other barriers that disproportionately impact underserved populations. Future studies should continue this work and build upon these strategies developed in CSER. Research is also needed to examine the relationship between direct or proxy measures of need for genomic medicine (e.g., expected prevalence of cancer susceptibility variants in a particular subpopulation) and measures of realized access (e.g., utilization of genetic counseling or screening tests).

#### Effective access to genomic medicine

Efforts like CSER are helping improve effective access to genomic medicine by expanding the evidence base and better characterizing access barriers. The effective access dimension focuses on improvements in health outcomes and clinical utility, aspects of which will be addressed in future CSER manuscripts [28]. Critically, accuracy of the interpretation of sequencing data depends upon the inclusion of ancestrally diverse populations in genomic research and assessing the impacts for these patients [4,12]. Thus, enrolling diverse populations in genomic research can also be considered to have social utility, resulting in accumulation of data and knowledge that can potentially improve clinical outcomes for various groups. Continued focus on developing a robust evidence base is in line with the ACMG’s recommendations that narrow definitions of “clinical utility” [86] should be broadened to include benefits that accrue outside of clinical care. Effective access also includes elements of patient satisfaction and health outcomes, which encompass the understudied topic of culturally competent and language concordant genomic communication. CSER has begun to fill these gaps through multiple avenues [27,87,88], including evaluating novel language-tailored genomics content and communication strategies like NYCKidSeq’s GUÍA tool [61]. Continued research utilizing stakeholder engagement and community-based participatory approaches [48,64,89,90] is needed to provide further insight into the experience of LEP populations, the impact of language barriers on quality of care, and the relationship between language and literacy barriers in genomics.

#### Efficient access to genomic medicine

Efficient access highlights a notion of technical efficiency, or value of genomics in terms of health outcomes per dollar spent. Tailoring management plans to individuals’ genetic makeup aligns with the goal of providing the right care for the right patient at the right time. Cost-effectiveness analyses are needed to determine whether some mix of less extensive and less costly genetic or nongenetic testing can achieve the same level of health as GS. Such analyses should consider the accuracy of genetic testing, actionability of results, cost-benefit balance of clinical and behavioral actions triggered by genetic testing results, and ease of modifying clinical processes of care to implement testing and downstream clinical process changes. Cost-effectiveness analyses can also help identify the value of specific genomic medicine applications for population subgroups, and newer methods incorporate equity impacts [91,92]. The burden on patients and families to obtain care, the quality of care received, and patients’ and families’ perspectives on the utility of testing should also be considered, since improved access to GS does not necessarily lead to clinical and social benefit, particularly for underrepresented and underserved populations. As it is still unclear how the current lower utility of GS for those with non-European ancestry may affect the idealized promise that genomics will eventually benefit all populations equitably, future genomic research should evaluate whether efforts to increase access to GS research and clinical care lead to the elimination of disparities in the utility of GS across ancestry groups. It is also important to consider trade-offs, as underserved communities may prioritize ensuring access to basic health care over increasing access to cutting-edge technologies like GS [93].

## Limitations

While this CSER assessment is theoretically and empirically informed, there are limitations to our approach. The number of persons who could benefit from but are not offered GS is unknown and we are only beginning to understand the reasons projects like those in CSER may fail to reach all eligible populations. Moreover, because we were studying access barriers in the clinical GS research context, we were unable to learn from individuals who face such significant barriers that they do not enroll in research. Most lead sites were large academic medical centers in urban areas, creating access barriers (e.g., transportation/travel) particularly for rural populations. Many of the academic medical centers involved in CSER functioned in a safety net treatment environment, and partnered with community-based sites and utilized telehealth strategies in an effort to address access barriers, but barriers undoubtedly remained. CSER survey measures also had shortcomings, including that the harmonized access to care measure did not capture language or sociocultural barriers. The measure used to assess health literacy, while validated in Spanish-speaking populations, may have lower specificity among Spanish-speaking groups as it does not consider respondents’ preferred language, English proficiency, or Spanish language variation [94]. As CSER’s work around effective access to genomic medicine was ongoing, results included are partial and this paper does not cover CSER’s community engagement efforts since these will be explored elsewhere. Finally, case studies were completed prior to the COVID-19 pandemic and a pre/post-pandemic comparison is beyond the scope of this paper. Research has begun to describe how the pandemic may negatively affect access to non-COVID-19 health research [95] and care [96], including genetic services [97]. Future research should investigate the extent to which the COVID-19 pandemic has exacerbated access barriers including challenges accessing telemedicine [98,99] and reaching traditionally marginalized populations such as individuals experiencing homelessness.

## Conclusion

We suggest adopting a broad conceptualization of access to care in the context of genomic research and genomic medicine that encompasses multiple dimensions, use of both quantitative and qualitative research methods to identify and explore access issues, and investment in the development and testing of innovative strategies to address these issues. This conceptualization can be applied to clinical research involving other cutting-edge technologies. Given our finding that GS research in the USA is a pathway for individuals to use services they would not otherwise have access to, we recommend including access to clinical GS research in any access to genomic medicine framework to better characterize the landscape of access-related factors. We also recommend consideration of access to needed nonmedical support services. Building on this work, the field has an opportunity and obligation to advance equitable, effective, and efficient access to genomic medicine both within and outside clinical research contexts.

## Supplementary material

For supplementary material accompanying this paper visit http://dx.doi.org/10.1017/cts.2021.855. click here to view supplementary material

## CSER Diversity and Inclusion Statement

In CSER, we aim to improve the use of genetic information in medicine and reduce barriers to genetic services among underserved groups. Our research seeks to better understand connections between genes, other drivers of health and disease, and health outcomes. We have worked with study participants and community partners to help make our research more inclusive. We still have much more work to do to ensure that our findings are applied in fair and just ways. We also acknowledge the need for more diversity among our own researchers. As we publish the results of CSER, we commit to carrying efforts forward to make sure people of all backgrounds benefit from genomic research and medicine.
