# Supplementary material for: Examining access to care in clinical genomic research and medicine: Experiences from the CSER Consortium
Source: J Clin Transl Sci. 2021 Sep 14;5(1):e193. doi: 10.1017/cts.2021.855 (PMC8634302; doi:10.1017/cts.2021.855)
Supplement: Supplementary file 1 [file S2059866121008554sup001.pdf]

Supplementary Material 1: Structuring Questions to Guide CSER Projects in Developing Case Studies.

Background

1. Please provide background information about your site and its particular access to care issues or considerations. For example, what percentage of your site's participants are uninsured? Does your state participate in the Medicaid expansion?

Experience with CSER Harmonized Measures

2. Please describe your experience to date using the CSER harmonized access to care measures (including the insurance and income question, although these are not labeled under the "access to care" domain).
    a. What kind of feedback, if any, has your site received from participants about these measures?
3. Please share any questions your site included as site-specific questions that were either included to ask about access to care, or that may be relevant to access to care even if that wasn't the primary purpose for including those questions.
4. In your preliminary data from access to care measures (harmonized or site-specific), have there been any interesting early findings? NOTE: This question is not intended to report data – we are more interested in what early data may indicate for how measures are functioning, if they came up in group discussions or impacted development of further measures.

Conceptual Development Experience

5. If relevant, what was the rationale behind the development and inclusion of site-specific questions that relate to access to care? Please describe how your research team conceptualizes how questions relate to access to care issues, how this factored into decision-making on what measures to use, and any notable conversations your research group had about measuring access to care.
6. What broader questions were raised either by designing data collection instruments for access to care, or in the process of data collection/interaction with participants? For example, the UCSF interviews prompted conversations about whether "access to care" encompasses only access to medical care or extends to access to medical care and social services related to a medical condition.
7. Please also include any other interesting thoughts, lessons learned, or experiences related to access to care that have come up at your site.

Supplementary Material 2: CSER Consortium Projects - Preliminary Data Relevant to Access to Care (Data through February 29, 2020 unless Otherwise Noted; All Adult/Parent Data unless Otherwise Noted).

**Study Name** (Lead Institution)	**CHARM** (Kaiser Permanente Northwest)	**KidsCanSeq** (Baylor College of Medicine)	**NCGENES 2** (University of North Carolina)	**NYCKidsSeq** (Icahn School of Medicine at Mount Sinai & Montefiore Medical Center)	**P3EGS** (University of California, San Francisco)	**SouthSeq**[a] (HudsonAlpha Institute for Biotechnology)
Insurance and Socioeconomic Status						
% (n) Uninsured	5.6% (41/738)	Parents: 35.3% (103/292)<br>Children: 5.8% (17/294)	10.8% (20/186)	Children: 0.2% (1/446)	0.01% (3/538)<br>- Pediatric: 0 (0/365)<br>- Prenatal: 1.7% (3/173)	Children: 2.6% (4/153)
% (n) with Medicaid/CHIP	17.8% (131/738)	Parents: 19.8% (38/192)<br>Children: 48.5% (144/297)	52.2% (97/186)	Children: 65.0% (288/446)	65.1% (350/538)<br>- Pediatric: 86.6% (316/365)<br>- Prenatal: 19.7% (34/173)	Children: 65.4% (100/153)
% (n) below 200% of the federal poverty line	37.2% (263/708)	52.0% (129/248)	57.3% (75/131)	47.3% (211/ 446)	48.7% (171/351)<br>- Pediatric: 66.8% (155/232)<br>- Prenatal: 13.4% (16/119)	57.1% (72/126)
HRSA medically underserved area status[b]	20.1% (165/793)	35.8% (108/302)	N/A	54.0% (241/446)	25.7% (138/538)<br>- Pediatric: 28.8% (105/365)	N/A

					- Prenatal: 19.1% (33/173)	
Health Literacy Scores[c]						
Limited (Lower literacy; %, n)	3.9% (31/793)	18.6% (55/296)	7.6% (10/131)	16.1% (72/446)	15.1% (69/457)<br>- Pediatric: 18.9% (57/302)<br>- Prenatal: 7.7% (12/155)	10.3% (16/156)
Marginal (Medium literacy; %, n)	14.6% (116/793)	70.6% (209/296)	29.0% (38/131)	21.7% (97/446)	22.8% (104/457)<br>- Pediatric: 25.2% (76/302)<br>- Prenatal: 18.1% (28/155)	25.6% (40/156)
Adequate (Higher literacy; %, n)	76.7% (608/793)	10.8% (32/296)	62.6% (82/131)	62.1% (277/446)	62.1% (284/457)<br>- Pediatric: 56.0% (169/302)<br>- Prenatal: 74.2% (115/155)	64.1% (100/156)
Language Proficiency and Preference						
% (n) Limited English proficient[d,e]	5.9% (43/735)<br>- English: 2.9% (19/665)<br>- Spanish: 34.3% (24/70)	5.9% (17/288)<br>- English: 4.0% (10/247)<br>- Spanish: 17.1% (7/41)	N/A	19.5% (87/446)<br>- English: 12.0% (42/350)<br>- Spanish:46.9% (45/96)	N/A - LEP data not collected	English[f]: 3.8% (6/159)
% (n) who prefer to speak a language other than English with doctors[e]	11.1% (27/243)<br>- English: 1.0% (2/208)<br>- Spanish: 71.4% (25/35)	2.1% (6/288)<br>- English: 0.4% (1/247)<br>- Spanish: 12.2% (5/41)	N/A	9.9% (44/446)<br>- English: 1.4% (5/350)<br>- Spanish: 40.6% (39/96)	28.8% (124/431)<br>- Pediatric: 36.0% (105/292)<br>- Prenatal: 13.7% (19/139)<br>- English: 4.9%	English[f]: 1.3% (2/159)

					(21/431)<br>- Pediatric: 5.5% (16/292)<br>- Prenatal: 3.6% (5/139)<br>- Spanish: 20.6% (89/431)<br>- Pediatric: 27.4% (80/292)<br>- Prenatal: 6.5% (9/139)	
Race/Ethnicity						
White/ European American	48.9% (367/751)	34.8% (102/293)	67.2% (88/131)	23.1% (99/429)	24.9% (124/498)<br>- Pediatric: 17.0% (57/336)<br>- Prenatal: 41.4% (67/162)	54.1% (85/157)
Hispanic/ Latino	26.9% (202/751)	44.7% (131/293)	1.5% (2/131)	43.4% (186/429)	41.6% (207/498)<br>- Pediatric: 51.8% (174/336)<br>- Prenatal: 20.4% (33/162)	2.5% (4/157)
Black or African American	4.9% (37/751)	9.2% (27/293)	14.5% (19/131)	16.3% (70/429)	4.0% (20/498)<br>- Pediatric: 4.8% (16/336)<br>- Prenatal: 2.5% (4/162)	32.5% (51/157)
Asian	4.7% (35/751)	4.1% (12/293)	3.8% (5/131)	7.5% (32/429)	12.0% (60/498)<br>- Pediatric: 9.3% (31/335)	0

					- Prenatal: 17.9% (29/162)	
American Indian, Native American, Alaska Native	1.7% (13/751)	0.7% (2/293)	3.1% (4/131)	0.5% (2/429)	0.8% (4/498)<br>- Pediatric: 1.2% (4/336)<br>- Prenatal: 0	0.6% (1/157)
Middle Eastern or North African/ Mediterranean	0.7% (5/751)	1.4% (4/293)	0	1.6% (7/429)	4.4% (22/498)<br>- Pediatric: 5.4% (18/336)<br>- Prenatal: 2.5% (4/162)	0.6% (1/157)
Native Hawaiian/ Pacific Islander	0.4% (3/751)	0.3% (1/293)	0	0	0.8% (4/498)<br>- Pediatric: 1.2% (4/336)<br>- Prenatal: 0	0
More than one	8.9% (67/751)	2.7% (8/293)	12.2% (16/131)	4.2% (18/429)	10.4% (52/498)<br>- Pediatric: 8.6% (29/336)<br>- Prenatal: 14.2% (23/162)	8.3% (13/157)
Prefer not to answer	1.3% (10/751)	1.4% (4/293)	0.8% (1/131)	1.9% (8/429)	0.2% (1/498)<br>- Pediatric: 0<br>- Prenatal: 0.6% (1/162)	0
Unknown/ None of these fully describe me	1.6% (12/751)	0.7% (2/293)	2.3% (3/131)	1.2% (5/429)	0.8% (4/498)<br>- Pediatric: 0.9% (3/336)<br>- Prenatal: 0.6% (1/162)	1.3% (2/157)

CSER: Clinical Sequencing Evidence-Generating Research consortium; NIH: National Institutes of Health; NHGRI: National Human Genome Research Institute; CHARM: Cancer Health Assessments Reaching Many; NCGENES 2: North Carolina Clinical Genomic Evaluation by Next-generation Exome Sequencing 2; P3EGS: Program in Prenatal and Pediatric Genome Sequencing.

[a]Includes data collected after February 29, 2020.

[b]Medically Underserved Areas and Populations (MUA/Ps). Bureau of Health Workforce. Published October 19, 2016. https://bhw.hrsa.gov/shortage-designation/muap.

[c]Source: Chew LD, Griffin JM, Partin MR, et al. Validation of Screening Questions for Limited Health Literacy in a Large VA Outpatient Population. J Gen Intern Med. 2008;23(5):561-566. doi:10.1007/s11606-008-0520-5.

[d]Limited English proficient (LEP) defined as speaking English less than "very well."

[e]If participant completed an English survey, they are categorized as “English preferred” for the study, while those who completed a Spanish survey are categorized as “Spanish preferred”; “English” and “Spanish” notation refers to if the responses are from those who took an English or Spanish survey.

[f]Not enrolling those whose preferred language is not English.
